# Transitioning Between Prostanoid Therapies in Pulmonary Arterial Hypertension

**DOI:** 10.3389/fmed.2020.00081

**Published:** 2020-03-31

**Authors:** Irene Z. Pan, Jessica R. Carey, Joshua A. Jacobs, John Dechand, Joshua J. Sessions, Teshia Sorensen, Brittany A. Penn, Jennalyn D. Mayeux, Nathan D. Hatton, John J. Ryan

**Affiliations:** ^1^Department of Pharmacy, University of Utah Health, Salt Lake City, UT, United States; ^2^Division of Cardiovascular Medicine, Department of Medicine, University of Utah, Salt Lake City, UT, United States; ^3^Division of Pulmonary and Critical Care Medicine, Department of Medicine, University of Utah, Salt Lake City, UT, United States

**Keywords:** pulmonary hypertension, therapeutics, right heart failure, pharmacology, prostaglandin, prostacyclin

## Abstract

**Background:** New oral prostacyclin therapies and prostacyclin agonists have become available for the treatment of pulmonary arterial hypertension (PAH). However, methods for transitioning between oral, inhaled, and parenteral formulations are not well-established, except in the form of case reports and case series. Collectively, these emphasize the lack of a standardized process and approach in transitioning patients between PAH prostanoid therapies. In this case series, we report our experience at an accredited Pulmonary Hypertension center in transitioning between various oral, inhaled, and parenteral prostanoids to offer additional guidance on safe transitions in therapy.

**Methods:** All cases of prostanoid transitions at an accredited Pulmonary Hypertension center from March 2018 to September 2019 were included in this report. The transition approach for each case was developed through a review of the literature, extrapolation of available pharmacokinetic data, and collaboration between pharmacists and clinicians.

**Results:** This case series describes the transition of 3 patients from selexipag to parenteral treprostinil; 1 patient transitioning from parenteral treprostinil to selexipag; 1 patient transitioning from oral treprostinil to parenteral treprostinil; and 1 patient transitioning from inhaled treprostinil to selexipag. Four of the 6 patients presented here were transitioned to an alternate prostanoid on account of clinical worsening, while the remaining 2 patients transitioned due to intolerance of parenteral therapy and poor medication adherence. This case series includes patients with various etiologies of PAH including idiopathic PAH, methamphetamine-associated PAH, and scleroderma-associated PAH. All patients successfully completed each transition without serious adverse events.

**Conclusions:** With the increasing utilization and availability of prostanoids, there is a critical need for a standardized approach in transitioning safely between different formulations without compromising treatment efficacy. In this case series, we present our clinical experiences, guided by available pharmacokinetic data, in transitioning between various prostanoid formulations.

## Background

In recent years, new oral prostanoid therapies such as selexipag and oral treprostinil have become globally available for the treatment of pulmonary arterial hypertension (PAH). These agents are becoming increasingly utilized due to their efficacy, oral route of administration, and potentially favorable side effect profile based on data from the FREEDOM trials with oral treprostinil (1–4) and the GRIPHON trial with selexipag (5). Established guidance on transitions between various formulations within this drug class have not been standardized. A number of case reports and case series have been published describing such transitions but lack consistent processes (6–13).

The most common method of determining dose equivalency between parenteral treprostinil and oral treprostinil is derived from a small phase 2 trial consisting of 33 patients. This dose conversion strategy can be found in the package insert for oral treprostinil and is summarized by the following equation (14):

Oral treprostinil total dose/day (mg) = parenteral treprostinil dose (ng/kg/min) × weight (kg) × 0.0072

During the transition period, the package insert recommends reducing parenteral treprostinil by up to 30 ng/kg/min per day while also increasing the dose of oral treprostinil by up to 6 mg per day as tolerated (14). However, despite sharing similar dosing strategies based on these recommendations, there are still numerous variations between different institutional practices. Most notable are the durations over which the transitions are implemented (6–10).

There is no standardized dose conversion strategy for transitioning from oral treprostinil to inhaled treprostinil, and no recommendations are provided in the package insert (14). There is published data for patients on doses of parenteral treprostinil ranging from 22.5 to 111 ng/kg/min and inhaled treprostinil ranging from 9 to 54 mcg four times daily, who have subsequently been transitioned to oral treprostinil 1 mg twice daily to 14 mg three times daily (7, 9, 10). Given the linear pharmacokinetic relationship between treprostinil plasma concentrations and doses up to 125 ng/kg/min, relative dose equivalencies between different formulations can be estimated (15). Parenteral treprostinil 10 ng/kg/min results in plasma concentrations of ~1–2 ng/mL, which is roughly equivalent to inhaled treprostinil 54 mcg four times daily (15–17). Assuming inhaled treprostinil 54 mcg four times daily is approximately equivalent to parenteral treprostinil 5–10 ng/kg/min, the corresponding dose of oral treprostinil (for an average 70 kg patient) would be ~1–2 mg three times daily. Other studies have described successful transitions between inhaled and parenteral treprostinil using parenteral doses of up to 25 ng/kg/min (18, 19).

For transitions between parenteral or inhaled prostanoids and selexipag, there is considerably less guidance available. Selexipag is an oral selective IP receptor agonist, and the pharmacokinetic relationship between dose and equivalent plasma concentrations are unknown. In the TRANSIT-1 study, 34 PAH patients on inhaled treprostinil were transitioned to selexipag over 8 weeks with a 94.1% success rate. The mean dose of inhaled treprostinil at baseline was 59.3 mcg four times daily (13). In a case series of 4 patients on parenteral treprostinil, the highest dose upon initiation of the transition was 46.5 ng/kg/min, which was successfully transitioned to selexipag 1,600 mcg twice daily (11). In another case series of 5 patients, parenteral treprostinil doses up to 96 ng/kg/min were successfully transitioned to selexipag 1,600 mcg twice daily (20). It is important to note the lack of information beyond 6 months following the transition, particularly in patients previously on high treprostinil doses.

In this case series, we report our experience in transitioning between oral, inhaled, and parenteral prostaglandin products at an accredited Pulmonary Hypertension (PH) center (Table 1).

**Table 1:** Baseline characteristics.

	**Case #1**	**Case #2**	**Case #3**	**Case #4**	**Case #5**	**Case #6**
Age (yr)	66	41	40	32	20	56
Type of PAH	Scleroderma-associated	Idiopathic	Methamphetamine-associated	Methamphetamine-associated	Idiopathic	Methamphetamine-associated
Transitioning agents	Selexipag to SQ Treprostinil	Selexipag to SQ Treprostinil	Selexipag to SQ Treprostinil	SQ Treprostinil to Selexipag	Oral Treprostinil to SQ Treprostinil	Inhaled Treprostinil to Selexipag
6MWD (m)	213	213	383	671	520	312
BNP (pg/mL)	288	579	NT-proBNP 599 pg/mL	21	43	189
Hemodynamics	RAP 25 mmHg PAP 61/26 mmHg mPAP 43 mmHg PCWP 9 mmHg PVR 6.7 WU CI 2.3 L/min/m^2^	RAP 19 mmHg PAP 80/40 mmHg mPAP 54 mmHg PCWP 18 mmHg PVR 8.6 WU CI 2.56 L/min/m^2^	RAP 5 mmHg PAP 87/32 mmHg mPAP 57 mmHg PCWP 12 mmHg PVR 9.3 WU CI 2.46 L/min/m^2^	RAP 3 mmHg PAP 120/57 mmHg mPAP 78 mmHg PCWP 17 mmHg PVR 9.2 WU CI 3.7 L/min/m^2^	RAP 10 mmHg PAP 100/55 mmHg mPAP 79 mmHg PCWP 21 mmHg PVR 13.68 WU CI 2.35 L/min/m^2^	RAP 6 mmHg PAP 63/25 mmHg mPAP 44 mmHg PCWP 7 mmHg PVR 11.7 WU CI 1.6 L/min/m^2^

## Methods

Approval of this study was obtained through the University of Utah Institutional Review Board. All cases that underwent transition between different prostanoid formulations at the University of Utah Pulmonary Hypertension Comprehensive Care Center from March 2018 to September 2019 were included in this report. No cases were omitted, and all available relevant data were included in this report. Written informed consent was obtained from the participants for the publication of this case report. The consecutive cases were collected prospectively. The different methodologies for transition were created after an extensive literature review and with collaboration between pharmacists and clinicians at University of Utah Health.

## Case Presentation

### Case #1: Selexipag to Parenteral Treprostinil

A 66-year-old female with World Health Organization (WHO) group 1, functional class (FC) III scleroderma-associated PAH underwent a planned admission to transition from selexipag to parenteral treprostinil due to declining clinical status. Prior to starting any PAH therapies, her right heart catheterization (RHC) showed a right atrial pressure (RAP) of 25 mmHg, pulmonary artery pressure (PAP) of 61/26 mmHg with a mean PAP of 43 mmHg, pulmonary capillary wedge pressure (PCWP) of 9 mmHg, pulmonary vascular resistance (PVR) of 6.7 Wood Units (WU), and cardiac index (CI) (Fick) of 2.3 L/min/m^2^. PAH medications prior to the transition were: ambrisentan 10 mg daily, sildenafil 20 mg three times daily, and selexipag 600 mcg twice daily. The patient had received selexipag for ~7 months. Her other medications included apixaban 5 mg twice daily, bumetanide 2 mg twice daily, and metolazone 2.5 mg twice weekly. Prior to the transition, while on PAH therapies, the patient had a 6 minute walk distance (6MWD) of 213 m; brain natriuretic peptide (BNP) of 288 pg/mL; and a transthoracic echocardiogram (TTE) demonstrating an estimated right ventricular systolic pressure (RVSP) of 61 mmHg, a moderate pericardial effusion, and normal right ventricle (RV) size and function. On admission, her total body weight was 101.4 kg (BMI 38.4 kg/m^2^). She was hemodynamically stable with an oxygen saturation of 99% on her baseline oxygen requirement of 1L. Selexipag total daily dose was decreased by 400 mcg per day (split between the morning and evening doses) starting on the day of hospital admission. Intravenous treprostinil was initiated at 5 ng/kg/min using an ideal body weight of 54.2 kg on day 1 of admission and increased by increments of 5 ng/kg/min every 24 hours to 10 ng/kg/min by the time of selexipag discontinuation. The transition was completed over 3 days (Table 2). The patient tolerated the transition well with no reports of headaches, flushing, flu-like symptoms, or symptomatic hypotension. Throughout the transition, she required 1 to 2 L/min of oxygen to maintain oxygen saturations >90% at rest. Her hospital stay was complicated by fluid overload and acute kidney injury likely secondary to cardiorenal syndrome, despite an adequate cardiac output shown on RHC. Following the transition, her RHC hemodynamics showed a RAP of 23 mmHg, PAP of 67/29 mmHg with a mean PAP of 44 mmHg, PCWP of 27 mmHg (increased PCWP secondary to right ventricular compression of the left ventricle), PVR of 2.2 WU, and CI (Fick) of 3.8 L/min/m^2^. Prior to hospital discharge, the patient was transitioned from IV to SQ treprostinil and was instructed to continue up-titrating by 1 ng/kg/min every 2–4 days until goal of 40 ng/kg/min. At a 7-month follow-up, she was on SQ treprostinil at a rate of 43 ng/kg/min. Her BNP was stable at 261 pg/mL, and her TTE was unchanged. However, her 6MWD had decreased to 168 m, and she required 3 L/min of oxygen to maintain an oxygen saturation of >88%. Repeat RHC showed a RAP of 19 mmHg, PAP of 61/25 mmHg with a mean PAP of 37 mmHg, PCWP of 22 mmHg, PVR of 1.7 WU, and CI (Fick) of 4.14 L/min/m^2^.

**Table 2:** Selexipag to parenteral treprostinil transitions (Cases #1-#3).

**Drug Titration**	**Case 1**	**Case 2**	**Case 3**
Selexipag dose prior to transition	600 mcg twice daily	1,600 mcg twice daily	1,400 mcg twice daily
Selexipag dose at time of treprostinil initiation	400 mcg	1,200 mcg	1,200 mcg
Treprostinil initiation dose	5 ng/kg/min	5 ng/kg/min	5 ng/kg/min
Selexipag dose down-titration	Total daily dose decreased by 400 mcg every 24 h	Total daily dose decreased by 800 mcg every 24 h until 400 mcg dose reached, then decreased by 400 mcg every 24 h	Decreased by 200 mcg per dose every 12 h
Treprostinil dose up-titration	Increased by 5 ng/kg/min every 24 h until selexipag off, then increased by 1 ng/kg/min every 24 h as tolerated	Increased by 3–5 ng/kg/min every 24 h until selexipag off, then increased by 1 ng/kg/min every 24 h as tolerated	Increased by 5 ng/kg/min every 24 h until selexipag off, then increased by 1 ng/kg/min every 24 h as tolerated
Treprostinil dosing weight used	54.2 kg (IBW)	70 kg (IBW)	50.1 kg (IBW)
Duration of transition	3 days	5 days	3 days
Treprostinil dose reached at hospital discharge	13 ng/kg/min	20 ng/kg/min	20 ng/kg/min

### Case #2: Selexipag to Parenteral Treprostinil

A 41-year-old female with WHO group 1, FC IV idiopathic PAH underwent a planned admission to transition from selexipag to parenteral treprostinil. She had worsening functional class on ambrisentan 10 mg daily, sildenafil 20 mg three times daily, and selexipag 1,600 mcg twice daily. Her concomitant medications included torsemide 40 mg daily, spironolactone 25 mg daily, digoxin 250 mcg daily, and apixaban 5 mg twice daily. Prior to the transition, while on PAH therapies, the patient had a 6MWD of 213 m, BNP of 579 pg/mL, and a TTE demonstrating an estimated RVSP of 69 mmHg and a severely enlarged RV with mildly reduced RV systolic function. Her RHC on admission showed a RAP of 19 mmHg, PAP of 80/40 mmHg with a mean PAP of 54 mmHg, PCWP of 18 mmHg, PVR of 8.6 WU, and CI (Fick) of 2.56 L/min/m^2^. On admission, her total body weight was 107 kg (BMI 44.6 kg/m^2^). She was hemodynamically stable with an oxygen saturation of 92% on her baseline oxygen requirement of 4L. Selexipag was reduced to 1,200 mcg twice daily (75% of her home dose) on the day of admission and subsequently decreased by 800 mcg per day (split between the morning and evening doses). Once 400 mcg was reached, the dose was further decreased to 200 mcg after 24 h before stopping. Intravenous treprostinil was initiated on hospital day 1 at 5 ng/kg/min using an ideal body weight of 70 kg and was increased by 3–5 ng/kg/min every 24 h to 20 ng/kg/min by the time of selexipag discontinuation. The transition took place over the course of 5 days (Table 2). The patient tolerated the transition with no complaints. The patient did develop a rash during the admission, which was thought to be contact dermatitis secondary to an adhesive. Throughout the transition, her oxygen saturations remained >88% on 4 to 6 L/min NC, and her renal function remained unchanged. Prior to discharge, the patient was transitioned from IV to SQ treprostinil. She was continued on a gradual up-titration as an outpatient with a goal of 40 ng/kg/min and had reached 30 ng/kg/min by the time of her 1 month follow up.

### Case #3: Selexipag to Parenteral Treprostinil

A 40-year-old female with WHO group 1, FC III methamphetamine-associated PAH underwent a planned admission for transition from oral selexipag to parenteral treprostinil. She initially presented to our PH clinic with progression of disease and worsening functional status despite treatment with tadalafil 40 mg daily, macitentan 10 mg daily, and selexipag 1,400 mcg twice daily for nearly 3 years. Her other medications included furosemide 40 mg in the morning and 20 mg in the afternoon and spironolactone 25 mg daily. Prior to her transition, the patient had a 6MWD of 382 m, NT-proBNP of 599 pg/mL, and a TTE revealing an estimated RVSP of 91 mmHg and a severely enlarged RV with mildly reduced RV systolic function. Her RHC showed a RAP of 5 mmHg, PAP of 87/32 mmHg with a mean PAP of 57 mmHg, PCWP of 12 mmHg, PVR of 9.3 WU, and CI (Fick) of 2.46 L/min/m^2^. On admission, her total body weight was 94.5 kg (BMI 38.1 kg/m^2^). She was hemodynamically stable with an oxygen saturation of 90% on her baseline oxygen requirement of 4L. Selexipag was decreased by 200 mcg every 12 h from her home dose of 1,400 mcg twice daily. IV treprostinil was initiated at 5 ng/kg/min using an ideal body weight of 50.1 kg at the time of the first selexipag dose decrease and subsequently uptitrated by 5 ng/kg/min every 24 h to 15 ng/kg/min by the time of selexipag discontinuation. The transition took place over a period of 3 days (Table 2). After selexipag discontinuation, treprostinil was increased to 20 ng/kg/min prior to hospital discharge. The patient tolerated the transition well with some mild nausea but had no other complaints. Her oxygen saturations remained >88% on 4–6 L/min of oxygen at night and on 1–3 L/min during the day throughout the transition. She was discharged on her home oxygen requirement of 4 L/min at night. On the day of discharge, the patient was transitioned from IV to SQ treprostinil with the plan to increase treprostinil by 3 ng/kg/min every 3–6 days to a goal of 35 ng/kg/min. At the time of publication, the patient is doing well but has not yet had follow up studies performed after her transition.

### Case #4: Parenteral Treprostinil to Selexipag

A 32-year-old male with WHO group 1, FC II methamphetamine-associated PAH was admitted for transition from subcutaneous treprostinil to oral selexipag. The reason for transition was patient preference as his quality of life had significantly declined due to being limited by daily pump management. This transition was strongly discouraged by his PH care team, but after several conversations regarding his perspective and the risks associated, he chose to transition with close follow up. The patient was diagnosed with PAH 10 months prior to admission and was originally started on epoprostenol at the time of diagnosis but was quickly transitioned to subcutaneous treprostinil. His dose of treprostinil at the time of this transition was 32 ng/kg/min using a dosing weight of 70.6 kg. He was also on tadalafil 40 mg daily and macitentan 10 mg daily. Prior to the transition, while on PAH therapies, the patient had a 6MWD of 671 m, BNP of 21 pg/ml, and a TTE with an RVSP of 71.6 mmHg with normal RV systolic function and mild enlargement. His most recent RHC measurements immediately prior to the transition demonstrated a RAP of 3 mmHg, PAP of 120/57 mmHg with a mean PAP of 78 mmHg, PCWP of 17 mmHg, PVR of 9.2 WU, and CI (Fick) of 3.7 L/min/m^2^. On admission, his total body weight was 65.5 kg (BMI 23.3 kg/m^2^). The patient was hemodynamically stable with an oxygen saturation of 96% on room air while on his home treprostinil, tadalafil, and macitentan. The patient underwent a simultaneous down-titration of treprostinil by 4 ng/kg/min and up-titration of selexipag by 200 mcg every 12 h. The transition took place over a period of 5 hospital days (Table 3). The patient had no complaints of side effects during the transition. The patient's renal function remained stable during the transition, and his oxygen saturation remained >88% on his baseline requirement of 3L nocturnal oxygen and room air throughout the day. The patient successfully reached 1,600 mcg BID at the end of the transition and was discharged home on tadalafil, macitentan, and selexipag. At a 3-month follow-up, the patient had a 6MWD of 649 m, a BNP of 29 pg/mL, and a TTE that demonstrated an RVSP of 94.4 mmHg and severe RV enlargement and normal RV systolic function. He had complaints of flushing and jaw pain on selexipag 1,600 mcg BID, and thus his dose was reduced to 1,400 mcg daily in the morning and 1,600 mcg daily at bedtime.

**Table 3:** Parenteral treprostinil to selexipag transition (Case #4).

	**Treprostinil dose titration**	**Selexipag dose titration**
Day 1 AM	32 ng/kg/min	0 mcg
Day 1 PM	28 ng/kg/min	200 mcg
Day 2 AM	24 ng/kg/min	400 mcg
Day 2 PM	20 ng/kg/min	600 mcg
Day 3 AM	16 ng/kg/min	800 mcg
Day 3 PM	12 ng/kg/min	1,000 mcg
Day 4 AM	8 ng/kg/min	1,200 mcg
Day 4 PM	4 ng/kg/min	1,400 mcg
Day 5 AM	0 ng/kg/min	1,600 mcg

### Case #5: Oral Treprostinil to Parenteral Treprostinil

A 20-year-old male with WHO group 1, FC III idiopathic PAH was admitted for transition from oral treprostinil to subcutaneous treprostinil. The patient had a diagnosis of PAH since the age of 4 and had been on PAH-specific therapies prior to presentation to our center. The reason for transition was declining functional status on tadalafil 40 mg daily, macitentan 10 mg daily, and treprostinil diolamine 4 mg three times daily. Other medications included aspirin 81 mg daily and digoxin 125 mcg daily. His 6MWD at baseline on PAH therapies was 520 m, with a desaturation to 69%, requiring 25L via nasal cannula to bring his oxygen saturation to 82% at the end of 6 minutes. His initial BNP was 43 pg/mL. His TTE showed an RVSP of 97.9 mmHg, moderate RV enlargement, severe RV wall thickness, and normal RV systolic function. His RHC immediately prior to his transition to IV treprostinil showed a RAP of 10 mmHg, PAP of 100/55 mmHg with a mean PAP of 79 mmHg, PCWP of 21 mmHg, PVR of 13.68 WU, and CI (Fick) of 2.35 L/min/m^2^. On admission, the patient's total body weight was 69.9 kg (BMI 23.4 kg/m^2^). The patient was hemodynamically stable on his home treprostinil and had an oxygen saturation of 90% on room air. The patient underwent a simultaneous down-titration of oral treprostinil by 1 mg three times daily every 24 h while initiating and increasing IV treprostinil by 6 ng/kg/min every 24 h. The transition took place over a period of 4 hospital days (Table 4). The patient had no complaints of side effects during the transition, and the patient's renal function remained stable during the transition. His oxygen saturation at rest remained >90% throughout the entire transition without supplemental oxygen and was 94% on room air at the time of discharge. The patient successfully reached 24 ng/kg/min at the end of the transition and was converted to subcutaneous treprostinil and discharged home without any problems. At 2-month follow-up, the patient was WHO-FC II and had reached a dose of 40 ng/kg/min. His 6MWD had improved to 613 m, but he still required 25L of oxygen to achieve an oxygen saturation of 83%. His treprostinil was gradually increased to 61 ng/kg/min. At his 6-month follow-up, there was no new RHC data, his BNP was stable at 52 pg/mL and a TTE demonstrated normal RV size, severe RV wall thickness, and normal RV function.

**Table 4:** Oral Treprostinil to Parenteral Treprostinil (Case #5).

	**Oral treprostinil down-titration**	**IV treprostinil dose titration**
Day 1 AM	4 mg	
Day 1 Mid-day	4 mg	6 ng/kg/min
Day 1 PM	3 mg	6 ng/kg/min
Day 2 AM	3 mg	6 ng/kg/min
Day 2 Mid-day	3 mg	12 ng/kg/min
Day 2 PM	2 mg	12 ng/kg/min
Day 3 AM	2 mg	12 ng/kg/min
Day 3 Mid-day	2 mg	18 ng/kg/min
Day 3 PM	1 mg	18 ng/kg/min
Day 4 AM	1 mg	18 ng/kg/min
Day 4 Mid-day	1 mg	24 ng/kg/min
Day 4 PM	OFF	24 ng/kg/min
	Discharged	

### Case #6: Inhaled Treprostinil to Selexipag

A 56-year-old male with WHO group 1 methamphetamine-associated PAH, diagnosed 3.5 years previously, presented to our PH clinic after being lost to follow-up for 15 months. At the time of reestablishing care, the patient was WHO-FC III and was directly admitted to the hospital for intravenous diuresis for right heart failure and re-initiation and optimization of PAH-specific therapies. The patient was experiencing intolerable cough and poor adherence to inhaled treprostinil, and the decision was made to transition him to selexipag. The patient was also non-adherent to a regimen of tadalafil 40 mg daily and macitentan 10 mg daily. His other medications included torsemide 80 mg daily, metolazone 5 mg as needed, potassium chloride 20 mEq three times daily, spironolactone 100 mg daily, and aspirin 81 mg daily. Prior to admission, the patient had a 6MWD of 312 m and resting oxygen saturation of 90% on room air. His initial BNP was 189 pg/mL. His TTE showed an RVSP of 65.9 mmHg, severe RV enlargement, and severely reduced RV systolic function. RHC showed a RAP of 6 mmHg, PAP of 63/25 mmHg with a mean PAP of 44 mmHg, PCWP of 7 mmHg, PVR of 11.7 WU, and CI (Fick) of 1.6 L/min/m^2^. On admission to the hospital, the patient was restarted on macitentan 10 mg daily, and treprostinil was changed to 6 puffs four times daily for medication optimization in anticipation of transitioning him from inhaled treprostinil to selexipag. He was WHO-FC II upon discharge from the hospital, but due to a delay in access had an interruption in macitentan for 6 days after discharge. The decision was then made to begin the transition from inhaled treprostinil to selexipag 2 weeks later as an outpatient. At the time of transition, the patient was using inhaled treprostinil 6 puffs three times daily. The plan was to initiate selexipag 200 mcg twice daily for 1 week while maintaining inhaled treprostinil at 6 puffs three times daily, then to continue the transition as outlined in Table 5. However, during the second week of the transition, the patient complained of increased cough related to inhaled treprostinil and wanted to discontinue use. The patient had also not increased selexipag as planned. The transition plan was modified as shown in Table 5. The patient reached a maintenance dose of 1,600 mcg twice daily approximately 6 weeks after starting selexipag. At a 6-month follow-up, he remained on selexipag 1,600 mcg twice daily. His 6MWD improved to 431 m, and his BNP was 164 pg/mL. His TTE at 8 months showed a reduction in RVSP to 53.4 mmHg, severe RV enlargement, and moderately to severely reduce RV function.

**Table 5:** Inhaled treprostinil to selexipag (Case #6).

**Planned transition**			**Actual transition**		
	**Inhaled treprostinil down-titration**	**Selexipag up-titration**		**Inhaled treprostinil down-titration**	**Selexipag up-titration**
Week 1	6 puffs three times daily	200 mcg twice daily	Week 1	6 puffs three times daily	200 mcg twice daily
Week 2	3 puffs three times daily	400 mcg twice daily	Week 2	3 puffs three times daily	400 mcg in the morning and 200 mcg in the evening
Week 3	OFF	600 mcg twice daily	Week 3	OFF	400 mcg twice daily
		Increase every 1–2 weeks as tolerated to max of 1,600 mcg twice daily			Increase every 1–2 weeks as tolerated to max of 1,600 mcg twice daily

## Discussion

These 6 cases provide real world experience of transitioning patients between oral, inhaled, and parenteral prostanoids at a specialized PAH center over an 18-month period. This series illustrates one of the many challenges in contemporary PAH management where there is a lack of consensus regarding transitions between different agents. As prescriptions for oral prostanoid products continue to rise and the number of specialized PAH centers and providers continue to expand, it is increasingly essential to share experiences and institutional practice patterns. The purpose of this case series was to provide additional insight into transition strategies and serve as a resource for other practitioners faced with the challenge of transitioning patients between alternative prostanoid formulations.

We created individualized approaches for transitioning between prostanoid formulations based on reviews of the available literature and extensive discussion amongst our PAH providers. The absence of standardized protocols to facilitate prostanoid transitions provides a challenge to those caring for PAH patients. The risks of providing a suboptimal dose with prostanoid transitions includes acute right ventricular failure and death. The risk of transitioning patients to significantly higher doses too quickly include hypotension resulting in organ failures, as well as significant side effects. This in turn may go on to limit the amount prescribed, thus leading to inadequate treatment of patients with this severe, progressive disease.

When faced with the clinical scenario of transitioning patients between various prostanoid agents, we found the limited number of published reports describing real-world experiences to be challenging. The goal of this paper is to serve as a practical guide to our approach and details the challenges associated with these transitions. This paper is distinct from prior reports because it is comprised of different strategies and methods of delivering prostanoids, namely oral, inhaled, intravenous, and subcutaneous. Prior reports largely focused on one type of transition.

Within this case series, the predominant reason for transitioning between prostanoid formulations was clinical deterioration. Less commonly, patients were transitioned due to intolerance or poor adherence to the previous delivery system. In addition to studying how best to safely implement transitions, it would also be worthwhile to further examine reasons to switch between different prostanoid analogs from both the patients' and providers' perspectives. In the era of shared-decision making, it is important to ensure patients have been thoroughly educated on initial prostanoid therapy options, and in turn, that transition between agents is feasible, as demonstrated in this case series (21).

More prostanoid analogs are currently under clinical investigation, including a dry powder inhaled form of treprostinil (22) and ralinepag (23), a novel prostacyclin IP receptor agonist. Unless efforts are made to study the optimum manner in which PAH providers and their patients can transition between agents, this area will become increasingly complex. It will be important for drug developers and those working in the field of PAH to study and develop clinical standards in transitioning between agents.

## Limitations

The cases presented here are small in number and come from a single center. Other reports have described more standardized protocols, with larger sample sizes and with some homogeneity between cases (7, 13).

In clinical practice there are well-known difficulties in categorizing patients with pulmonary hypertension. For example, in this series, several patients displayed a PCWP >15 mmHg at various time points during their clinical care. This reflects the difficulty with interpreting hemodynamics in PAH patients and emphasizes one of the challenges in PH management. All patients met WHO group I PAH criteria at the time of therapy initiation. However, over time, several patients developed progressive heart failure with elevated PCWP likely due to left ventricular compression from an oversized right ventricle. As is routine in our and other PH programs, these cases are reviewed thoroughly and repeatedly in order to have a confirmatory diagnosis of PAH.

During the period under study, no patients were transitioned from or to epoprostenol. Therefore, such transitions are not presented in this paper. Although our transitions were well-tolerated clinically, it remains difficult to compare approaches between different institutions.

## Conclusions

With the increased use of oral prostanoids, there is a critical need to provide evidence- and pharmacokinetic-based guidance on how to transition between oral and parenteral prostanoids. In this case series, we present our approach for this practice in the hope to generate discussion and to determine best practice in this area.

## Ethics Statement

Written informed consent was obtained from the participants for the publication of this case report.

## Author Contributions

All authors contributed to reviewing the source data and crafting the manuscript.

### Conflict of Interest

The authors declare that the research was conducted in the absence of any commercial or financial relationships that could be construed as a potential conflict of interest.
